# 1-[2-Oxo-5-(trifluoro­meth­oxy)indolin-3-yl­idene]-4-[4-(trifluoro­methyl)­phen­yl]thio­semicarbazide

**DOI:** 10.1107/S1600536810023494

**Published:** 2010-06-23

**Authors:** Humayun Pervez, Mohammad S. Iqbal, Naveeda Saira, Muhammad Yaqub, M. Nawaz Tahir

**Affiliations:** a Department of Chemistry, Bahauddin Zakariya University, Multan 60800, Pakistan; b Department of Chemistry, Government College University, Lahore, Pakistan; c Department of Physics, University of Sargodha, Sargodha, Pakistan

## Abstract

In the title compound, C_17_H_10_F_6_N_4_O_2_S, an intra­molecular N—H⋯N hydrogen bonds forms an *S*(5) ring whereas N—H⋯O and C—H⋯S inter­actions complete *S*(6) ring motifs. The dihedral angle between the fused ring system and the phenyl ring is 6.68 (8)°. In the crystal, the mol­ecules are dimerized due to N—H⋯O inter­actions. π–π inter­actions are present between the benzene rings [centroid–centroid distance = 3.6913 (15) Å] and between the five membered ring and the trifluoro­meth­yl)phenyl ring [centroids–centroid distance = 3.7827 (16) Å]. One of the trifluoro­meth­oxy F atoms is disordered over two sites with occupancy ratio of 0.76 (3):0.24 (3). The F atoms of the *p*-trifluoro­methyl substituent are disordered over three sets of sites with an occupancy ratio of 0.70 (2):0.152 (11):0.147 (13).

## Related literature

For background to the synthesis, see: Pervez *et al.* (2009 ▶, 2010*b* ▶,*c* ▶). For a related structure, see: Pervez *et al.* (2010*a* ▶). For graph-set notation, see: Bernstein *et al.* (1995 ▶).
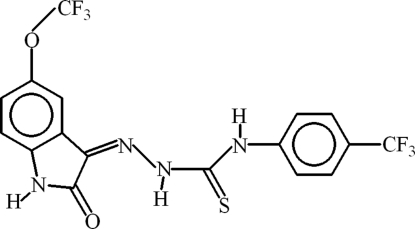


## Experimental

### 

#### Crystal data

C_17_H_10_F_6_N_4_O_2_S
*M* *_r_* = 448.35
Triclinic, 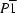

*a* = 7.5452 (11) Å
*b* = 8.3177 (13) Å
*c* = 16.048 (2) Å
α = 104.452 (6)°
β = 94.752 (7)°
γ = 103.606 (7)°
*V* = 937.1 (2) Å^3^
*Z* = 2
Mo *K*α radiation
μ = 0.25 mm^−1^
*T* = 296 K
0.32 × 0.24 × 0.22 mm

#### Data collection

Bruker Kappa APEXII CCD diffractometer
Absorption correction: multi-scan (*SADABS*; Bruker, 2005 ▶) *T*_min_ = 0.942, *T*_max_ = 0.952
13964 measured reflections
3351 independent reflections
2191 reflections with *I* > 2σ(*I*)
*R*_int_ = 0.045

#### Refinement

*R*[*F* ^2^ > 2σ(*F* ^2^)] = 0.043
*wR*(*F* ^2^) = 0.113
*S* = 1.02
3351 reflections
302 parameters
11 restraints
H-atom parameters constrained
Δρ_max_ = 0.24 e Å^−3^
Δρ_min_ = −0.24 e Å^−3^

### 

Data collection: *APEX2* (Bruker, 2007 ▶); cell refinement: *SAINT* (Bruker, 2007 ▶); data reduction: *SAINT*; program(s) used to solve structure: *SHELXS97* (Sheldrick, 2008 ▶); program(s) used to refine structure: *SHELXL97* (Sheldrick, 2008 ▶); molecular graphics: *ORTEP-3 for Windows* (Farrugia, 1997 ▶) and *PLATON* (Spek, 2009 ▶); software used to prepare material for publication: *WinGX* (Farrugia, 1999 ▶) and *PLATON*.

## Supplementary Material

Crystal structure: contains datablocks global, I. DOI: 10.1107/S1600536810023494/si2270sup1.cif

Structure factors: contains datablocks I. DOI: 10.1107/S1600536810023494/si2270Isup2.hkl

Additional supplementary materials:  crystallographic information; 3D view; checkCIF report

## Figures and Tables

**Table 1:** Hydrogen-bond geometry (Å, °)

*D*—H⋯*A*	*D*—H	H⋯*A*	*D*⋯*A*	*D*—H⋯*A*
N1—H1⋯O1^i^	0.86	1.98	2.829 (3)	168
N3—H3*A*⋯O1	0.86	2.01	2.716 (3)	138
N4—H4*A*⋯N2	0.86	2.19	2.627 (3)	111
C12—H12⋯S1	0.93	2.56	3.210 (3)	128

Symmetry code: (i) 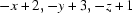.

**Fig. 1.**
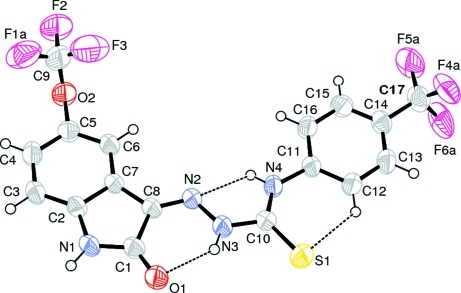
View of the title compound with the atom numbering scheme. The thermal ellipsoids are drawn at the 50% probability level. H-atoms are shown by small circles of arbitrary radii. The dotted lines indicate the intra-molecular H-bondings. Only the majority group of F-atoms are shown for clarity.

**Fig. 2.**
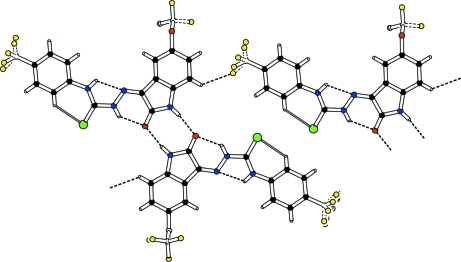
The partial packing (PLATON; Spek, 2009) which shows that molecules are dimerized and interlinked.

## supplementary crystallographic information

### Comment

In continuation of our earlier studies on the synthesis of biologically important *N*^4^-arylsubstituted isatins-3-thiosemicarbazones (Pervez *et al.*, 2009, 2010*b*, 2010*c*), here we report the synthesis and crystal structure of the title compound (I, Fig. 1).

The crystal structure of (II) *i.e.* 4-(5-chloro-2-methylphenyl)-1-(2-oxo-5-(trifluoromethoxy) indolin-3-ylidene) thiosemicarbazide has been published (Pervez *et al.*, 2010*a*). The title compound (I) differs from (II) due to the presence of trifluoromethyl group at position-4 instead of methyl and chloro functions at position-2 and -5, respectively, of the phenyl ring substituted at N^4^- of the thiosemicarbazone moiety.

In (I), the 2-oxoindolin A (C1–C8/N1/O1), thiosemicarbazone moiety B (N2/N3/C10/S1/N4) and the phenyl ring C (C11—C16) having *p*-trifluoromethyl function are planar with r. m. s. deviations of 0.0402, 0.0184 and 0.0119 Å, respectively. The dihedral angle between A/B, A/C and B/C is 6.78 (9), 6.68 (8) and 13.42 (10)°, respectively. Due to intramolecular H-bondings (Table 1, Fig. 1), one S(5) and two S(6) (Bernstein *et al.*, 1995) ring motifs are formed. The molecules are dimerized (Fig. 2) due to intermolecular H-bonding of N—H···O type with *R*_2_^2^(8) ring motifs. The dimers are interlinked through C—H···F type of H-bonding. There exist π···π interaction at a distance of 3.6913 (15) Å between the centroids of phenyl rings (C2—C7) and (C11—C16). Similarly, π···π interaction between the centroids of the heterocyclic ring (N1/C1/C8/C7/C2) and the phenyl ring (C11—C16) is 3.7827 (16) Å.

One of the F-atom of trifluoromethoxy group is disordered over two set of sites with occupancy ratio of 0.76 (3):0.24 (3). The F-atoms of *p*-trifluoromethyl function are disordered over three groups with occupancy ratio of 0.70 (2):0.152 (11):0.147 (13).

### Experimental

4-(4-Trifluoromethylphenyl)thiosemicarbazide (0.94 g, 4.0 mmol) dissolved in ethanol (10 ml) was added to a hot solution of 5-(trifluoromethoxy)indolin-2,3-dione (0.92 g, 4.0 mmol) in 50% aqueous ethanol (20 ml) containing a catalytic quantity of glacial acetic acid. The reaction mixture was then refluxed for 2 h. The yellow powder formed during refluxing was collected by suction filtration. Thorough washing with hot aqueous ethanol afforded the title compound (I) in pure form (1.34 g, 75%), m.p. 513 K. The yellow crystals of the title compound for *x*-ray analysis were obtained from the solution of ethyl acetate-petroleum ether (2:5) at room temperature by diffusion method.

### Refinement

The refinement dictated that only one F-atom of trifluoromethoxy group and all F-atoms of *p*-trifluoromethyl function are disordered. The best result is obtained if F-atom of trifluoromethoxy group is refined over two set of sites with occupancy ratio of 0.76 (3):0.24 (3) with equal anisotropic thermal parameters. Similarly to get the best result F-atoms of *p*-trifluoromethyl function are treated disordered over three set of sites with occupancy ratio of 0.70 (2):0.152 (11):0.147 (13). In these sets, the minor groups are treated anisotropically with equal thermal parameters and the major group as anisotropic having different thermal parameters.

The H-atoms were positioned geometrically (N–H = 0.86 Å, C–H = 0.93 Å) and refined as riding with *U*_iso_(H) = *xU*_eq_(C, N), where *x* = 1.2 for all H-atoms.

### Crystal data

**Table:**

C_17_H_10_F_6_N_4_O_2_S	*Z* = 2
*M**_r_* = 448.35	*F*(000) = 452
Triclinic, *P*1	*D*_x_ = 1.589 Mg m^−^^3^
Hall symbol: -P 1	Mo *K*α radiation, λ = 0.71073 Å
*a* = 7.5452 (11) Å	Cell parameters from 2191 reflections
*b* = 8.3177 (13) Å	θ = 2.6–25.3°
*c* = 16.048 (2) Å	µ = 0.25 mm^−^^1^
α = 104.452 (6)°	*T* = 296 K
β = 94.752 (7)°	Prism, yellow
γ = 103.606 (7)°	0.32 × 0.24 × 0.22 mm
*V* = 937.1 (2) Å^3^	

### Data collection

**Table:**

Bruker Kappa APEXII CCD diffractometer	3351 independent reflections
Radiation source: fine-focus sealed tube	2191 reflections with *I* > 2σ(*I*)
graphite	*R*_int_ = 0.045
Detector resolution: 8.2 pixels mm^-1^	θ_max_ = 25.3°, θ_min_ = 2.6°
ω scans	*h* = −9→6
Absorption correction: multi-scan (*SADABS*; Bruker, 2005)	*k* = −9→9
*T*_min_ = 0.942, *T*_max_ = 0.952	*l* = −19→19
13964 measured reflections	

### Refinement

**Table:**

Refinement on *F*^2^	Primary atom site location: structure-invariant direct methods
Least-squares matrix: full	Secondary atom site location: difference Fourier map
*R*[*F*^2^ > 2σ(*F*^2^)] = 0.043	Hydrogen site location: inferred from neighbouring sites
*wR*(*F*^2^) = 0.113	H-atom parameters constrained
*S* = 1.02	*w* = 1/[σ^2^(*F*_o_^2^) + (0.0501*P*)^2^ + 0.1774*P*] where *P* = (*F*_o_^2^ + 2*F*_c_^2^)/3
3351 reflections	(Δ/σ)_max_ = 0.001
302 parameters	Δρ_max_ = 0.24 e Å^−^^3^
11 restraints	Δρ_min_ = −0.24 e Å^−^^3^

### Special details

**Table:**

Geometry. Bond distances, angles *etc*. have been calculated using the rounded fractional coordinates. All su's are estimated from the variances of the (full) variance-covariance matrix. The cell e.s.d.'s are taken into account in the estimation of distances, angles and torsion angles
Refinement. Refinement of *F*^2^ against ALL reflections. The weighted *R*-factor *wR* and goodness of fit *S* are based on *F*^2^, conventional *R*-factors *R* are based on *F*, with *F* set to zero for negative *F*^2^. The threshold expression of *F*^2^ > σ(*F*^2^) is used only for calculating *R*-factors(gt) *etc*. and is not relevant to the choice of reflections for refinement. *R*-factors based on *F*^2^ are statistically about twice as large as those based on *F*, and *R*- factors based on ALL data will be even larger.

### Fractional atomic coordinates and isotropic or equivalent isotropic displacement parameters (Å^2^)

**Table:**

	*x*	*y*	*z*	*U*_iso_*/*U*_eq_	Occ. (<1)
S1	0.59532 (11)	0.73584 (9)	0.47879 (5)	0.0642 (3)	
F1A	0.786 (2)	1.3866 (16)	−0.0060 (4)	0.113 (2)	0.76 (3)
F2	0.5218 (3)	1.2388 (3)	−0.08045 (11)	0.1050 (9)	
F3	0.6805 (3)	1.1403 (3)	−0.00024 (13)	0.1212 (11)	
F4A	0.1431 (7)	−0.0888 (4)	0.1803 (6)	0.091 (2)	0.70 (2)
F5A	−0.0432 (14)	−0.0301 (5)	0.0936 (4)	0.110 (3)	0.70 (2)
F6A	−0.1071 (10)	−0.0475 (4)	0.2190 (6)	0.107 (2)	0.70 (2)
O1	0.8661 (2)	1.2725 (2)	0.48040 (11)	0.0593 (7)	
O2	0.5300 (3)	1.3270 (2)	0.05819 (12)	0.0636 (7)	
N1	0.8671 (3)	1.4593 (3)	0.39550 (13)	0.0496 (7)	
N2	0.5938 (3)	1.0294 (2)	0.32681 (12)	0.0455 (7)	
N3	0.6239 (3)	0.9659 (3)	0.39467 (13)	0.0512 (7)	
N4	0.4128 (3)	0.7152 (2)	0.32184 (13)	0.0496 (7)	
C1	0.8144 (3)	1.3071 (3)	0.41361 (16)	0.0466 (9)	
C2	0.7898 (3)	1.4494 (3)	0.31108 (16)	0.0446 (8)	
C3	0.8150 (3)	1.5752 (3)	0.26892 (17)	0.0536 (9)	
C4	0.7282 (3)	1.5321 (3)	0.18411 (18)	0.0565 (10)	
C5	0.6218 (3)	1.3664 (3)	0.14478 (16)	0.0502 (9)	
C6	0.5918 (3)	1.2393 (3)	0.18672 (15)	0.0469 (8)	
C7	0.6774 (3)	1.2829 (3)	0.27192 (15)	0.0414 (8)	
C8	0.6832 (3)	1.1869 (3)	0.33561 (15)	0.0423 (8)	
C9	0.6260 (5)	1.2776 (5)	−0.0049 (2)	0.0792 (14)	
C10	0.5363 (3)	0.8008 (3)	0.39415 (15)	0.0454 (8)	
C11	0.3131 (3)	0.5405 (3)	0.29085 (16)	0.0446 (8)	
C12	0.2921 (4)	0.4258 (3)	0.34125 (17)	0.0564 (9)	
C13	0.1977 (4)	0.2559 (3)	0.30334 (18)	0.0588 (10)	
C14	0.1225 (3)	0.1981 (3)	0.21696 (18)	0.0523 (9)	
C15	0.1373 (3)	0.3133 (3)	0.16787 (18)	0.0579 (9)	
C16	0.2315 (3)	0.4828 (3)	0.20459 (17)	0.0543 (9)	
C17	0.0301 (5)	0.0118 (4)	0.1773 (2)	0.0696 (13)	
F6B	−0.128 (2)	−0.001 (2)	0.1300 (17)	0.084 (4)	0.152 (11)
F6C	0.055 (4)	−0.041 (2)	0.0970 (13)	0.084 (4)	0.147 (13)
F4C	−0.1510 (19)	−0.023 (3)	0.1788 (19)	0.084 (4)	0.147 (13)
F1B	0.727 (4)	1.432 (4)	−0.0021 (14)	0.113 (2)	0.24 (3)
F4B	−0.023 (4)	−0.061 (2)	0.2404 (11)	0.084 (4)	0.152 (11)
F5B	0.143 (3)	−0.071 (2)	0.138 (2)	0.084 (4)	0.152 (11)
F5C	0.096 (3)	−0.087 (2)	0.2206 (18)	0.084 (4)	0.147 (13)
H3A	0.70052	1.03089	0.43980	0.0615*	
H15	0.08340	0.27634	0.10976	0.0695*	
H16	0.24060	0.55988	0.17100	0.0652*	
H4	0.74155	1.61460	0.15357	0.0679*	
H4A	0.39115	0.77794	0.28900	0.0595*	
H6	0.51745	1.12914	0.15911	0.0563*	
H12	0.34120	0.46323	0.40001	0.0676*	
H13	0.18465	0.17879	0.33702	0.0704*	
H1	0.93911	1.55096	0.43112	0.0594*	
H3	0.88805	1.68594	0.29640	0.0643*	

### Atomic displacement parameters (Å^2^)

**Table:**

	*U*^11^	*U*^22^	*U*^33^	*U*^12^	*U*^13^	*U*^23^
S1	0.0831 (5)	0.0563 (5)	0.0494 (4)	0.0101 (4)	−0.0045 (4)	0.0202 (3)
F1A	0.091 (5)	0.147 (5)	0.0733 (15)	−0.016 (4)	0.027 (2)	0.021 (2)
F2	0.1342 (17)	0.1161 (16)	0.0486 (11)	0.0132 (13)	−0.0175 (11)	0.0230 (11)
F3	0.178 (2)	0.136 (2)	0.0743 (14)	0.0873 (18)	0.0274 (13)	0.0277 (13)
F4A	0.105 (3)	0.0511 (18)	0.113 (5)	0.0310 (18)	0.026 (3)	0.004 (2)
F5A	0.134 (8)	0.064 (2)	0.089 (3)	−0.012 (3)	−0.033 (4)	−0.002 (2)
F6A	0.086 (4)	0.0480 (19)	0.175 (6)	−0.009 (2)	0.059 (4)	0.023 (3)
O1	0.0684 (12)	0.0488 (11)	0.0473 (11)	−0.0009 (9)	−0.0120 (9)	0.0116 (9)
O2	0.0700 (12)	0.0651 (13)	0.0498 (12)	0.0093 (10)	−0.0072 (10)	0.0186 (10)
N1	0.0535 (12)	0.0370 (12)	0.0440 (13)	−0.0028 (10)	−0.0063 (10)	0.0041 (10)
N2	0.0528 (12)	0.0378 (12)	0.0411 (12)	0.0051 (10)	0.0037 (9)	0.0097 (10)
N3	0.0637 (13)	0.0373 (12)	0.0429 (12)	0.0005 (10)	−0.0051 (10)	0.0099 (10)
N4	0.0601 (13)	0.0369 (12)	0.0454 (13)	0.0016 (10)	−0.0052 (10)	0.0149 (10)
C1	0.0455 (14)	0.0414 (15)	0.0454 (16)	0.0046 (12)	−0.0004 (12)	0.0074 (12)
C2	0.0440 (13)	0.0374 (14)	0.0451 (15)	0.0033 (11)	0.0033 (11)	0.0063 (12)
C3	0.0578 (16)	0.0373 (14)	0.0562 (18)	0.0004 (12)	0.0018 (13)	0.0098 (13)
C4	0.0645 (17)	0.0438 (16)	0.0597 (18)	0.0063 (13)	0.0051 (14)	0.0204 (14)
C5	0.0522 (15)	0.0489 (16)	0.0448 (16)	0.0082 (13)	−0.0023 (12)	0.0125 (13)
C6	0.0493 (14)	0.0387 (14)	0.0456 (15)	0.0047 (11)	0.0006 (12)	0.0077 (12)
C7	0.0415 (13)	0.0363 (13)	0.0399 (14)	0.0037 (11)	0.0015 (10)	0.0067 (11)
C8	0.0432 (13)	0.0363 (14)	0.0402 (14)	0.0036 (11)	0.0006 (10)	0.0061 (11)
C9	0.102 (3)	0.077 (2)	0.051 (2)	0.007 (2)	−0.0007 (19)	0.0236 (18)
C10	0.0521 (14)	0.0402 (14)	0.0410 (15)	0.0097 (12)	0.0049 (12)	0.0092 (12)
C11	0.0472 (14)	0.0373 (14)	0.0460 (15)	0.0052 (11)	0.0046 (11)	0.0120 (12)
C12	0.0705 (17)	0.0460 (16)	0.0491 (16)	0.0049 (14)	0.0042 (13)	0.0185 (13)
C13	0.0663 (17)	0.0435 (16)	0.066 (2)	0.0047 (14)	0.0098 (15)	0.0235 (14)
C14	0.0462 (14)	0.0398 (15)	0.0637 (18)	0.0012 (12)	0.0083 (13)	0.0111 (14)
C15	0.0589 (16)	0.0521 (17)	0.0497 (16)	−0.0004 (13)	−0.0030 (13)	0.0086 (14)
C16	0.0620 (16)	0.0464 (16)	0.0490 (16)	0.0010 (13)	−0.0004 (13)	0.0186 (13)
C17	0.069 (2)	0.0494 (18)	0.081 (3)	0.0013 (17)	0.0123 (19)	0.0147 (17)
F6B	0.080 (6)	0.053 (4)	0.097 (8)	0.003 (3)	0.026 (5)	−0.007 (4)
F6C	0.080 (6)	0.053 (4)	0.097 (8)	0.003 (3)	0.026 (5)	−0.007 (4)
F4C	0.080 (6)	0.053 (4)	0.097 (8)	0.003 (3)	0.026 (5)	−0.007 (4)
F1B	0.091 (5)	0.147 (5)	0.0733 (15)	−0.016 (4)	0.027 (2)	0.021 (2)
F4B	0.080 (6)	0.053 (4)	0.097 (8)	0.003 (3)	0.026 (5)	−0.007 (4)
F5B	0.080 (6)	0.053 (4)	0.097 (8)	0.003 (3)	0.026 (5)	−0.007 (4)
F5C	0.080 (6)	0.053 (4)	0.097 (8)	0.003 (3)	0.026 (5)	−0.007 (4)

### Geometric parameters (Å, °)

**Table:**

S1—C10	1.648 (3)	N3—H3A	0.8600
F1A—C9	1.333 (15)	N4—H4A	0.8600
F1B—C9	1.32 (3)	C1—C8	1.500 (3)
F2—C9	1.311 (4)	C2—C7	1.399 (4)
F3—C9	1.318 (5)	C2—C3	1.367 (4)
F4A—C17	1.334 (6)	C3—C4	1.381 (4)
F4B—C17	1.346 (18)	C4—C5	1.380 (4)
F4C—C17	1.332 (17)	C5—C6	1.375 (4)
F5A—C17	1.336 (7)	C6—C7	1.384 (3)
F5B—C17	1.32 (2)	C7—C8	1.449 (3)
F5C—C17	1.35 (2)	C11—C16	1.383 (4)
F6A—C17	1.342 (8)	C11—C12	1.387 (4)
F6B—C17	1.33 (2)	C12—C13	1.378 (4)
F6C—C17	1.30 (2)	C13—C14	1.372 (4)
O1—C1	1.233 (3)	C14—C15	1.376 (4)
O2—C9	1.331 (4)	C14—C17	1.489 (4)
O2—C5	1.423 (3)	C15—C16	1.373 (4)
N1—C1	1.346 (4)	C3—H3	0.9300
N1—C2	1.405 (3)	C4—H4	0.9300
N2—N3	1.349 (3)	C6—H6	0.9300
N2—C8	1.291 (3)	C12—H12	0.9300
N3—C10	1.374 (4)	C13—H13	0.9300
N4—C10	1.348 (3)	C15—H15	0.9300
N4—C11	1.410 (3)	C16—H16	0.9300
N1—H1	0.8600		
			
S1···N1^i^	3.524 (3)	N3···O1	2.716 (3)
S1···C12	3.210 (3)	N4···N2	2.627 (3)
S1···C13^ii^	3.687 (3)	N2···H4A	2.1900
S1···C1^iii^	3.652 (3)	C1···C13^vi^	3.568 (4)
S1···C12^ii^	3.597 (3)	C1···S1^iii^	3.652 (3)
S1···H12	2.5600	C3···F6A^xii^	3.362 (5)
S1···H12^ii^	2.9300	C3···F4B^xii^	3.147 (18)
S1···H13^ii^	3.1000	C3···C16^vi^	3.579 (3)
F1A···C4	3.097 (8)	C3···C10^xi^	3.558 (3)
F1A···F5B^iv^	3.12 (3)	C4···F6A^xii^	3.309 (5)
F1B···C4	2.89 (2)	C4···F4B^xii^	3.316 (19)
F2···F4C^v^	3.01 (2)	C4···F1A	3.097 (8)
F2···F6B^v^	3.071 (17)	C4···F1B	2.89 (2)
F3···C6	3.080 (3)	C5···C16^xi^	3.446 (3)
F3···F6B^vi^	3.06 (2)	C6···F6B^vi^	3.270 (17)
F3···F5B^iv^	2.68 (3)	C6···C14^xi^	3.562 (3)
F3···F6C^iv^	2.78 (3)	C6···F4C^vi^	3.23 (2)
F4B···C3^vii^	3.147 (18)	C6···F3	3.080 (3)
F4B···C4^vii^	3.316 (19)	C7···F4C^vi^	3.22 (2)
F4C···C8^viii^	3.21 (3)	C7···C12^xi^	3.550 (4)
F4C···C6^viii^	3.23 (2)	C8···F6A^vi^	3.151 (8)
F4C···C7^viii^	3.22 (2)	C8···F4C^vi^	3.21 (3)
F4C···N2^viii^	3.19 (2)	C9···F5B^iv^	3.28 (3)
F4C···F2^v^	3.01 (2)	C10···C3^i^	3.558 (3)
F5B···F1A^iv^	3.12 (3)	C12···C7^i^	3.550 (4)
F5B···F3^iv^	2.68 (3)	C12···S1	3.210 (3)
F5B···C9^iv^	3.28 (3)	C12···S1^ii^	3.597 (3)
F6A···C3^vii^	3.362 (5)	C12···N1^viii^	3.447 (4)
F6A···C8^viii^	3.151 (8)	C13···S1^ii^	3.687 (3)
F6A···N2^viii^	3.045 (8)	C13···C1^viii^	3.568 (4)
F6A···C4^vii^	3.309 (5)	C14···C6^i^	3.562 (3)
F6B···F2^v^	3.071 (17)	C16···C5^i^	3.446 (3)
F6B···C6^viii^	3.270 (17)	C16···C3^viii^	3.579 (3)
F6B···F3^viii^	3.06 (2)	C1···H1^x^	2.7900
F6C···F3^iv^	2.78 (3)	C1···H3A	2.4100
F1A···H16^ix^	2.7900	C10···H12	2.8800
F1B···H4	2.5600	H1···O1^x^	1.9800
F1B···H16^ix^	2.7600	H1···C1^x^	2.7900
F4A···H13	2.8500	H3···F4B^xii^	2.4600
F4B···H3^vii^	2.4600	H3···F6A^xii^	2.7900
F4B···H4^vii^	2.8000	H3···F5C^xii^	2.7500
F4B···H13	2.3300	H3A···C1	2.4100
F4C···H4^vii^	2.8500	H3A···O1	2.0100
F5A···H15	2.4400	H4···F1B	2.5600
F5B···H6^i^	2.8700	H4···F6A^xii^	2.6700
F5C···H13	2.4200	H4···F4B^xii^	2.8000
F5C···H3^vii^	2.7500	H4···F4C^xii^	2.8500
F6A···H3^vii^	2.7900	H4A···N2	2.1900
F6A···H13	2.7500	H4A···H16	2.2600
F6A···H4^vii^	2.6700	H6···F5B^xi^	2.8700
F6B···H15	2.5900	H12···C10	2.8800
F6C···H15	2.5500	H12···S1	2.5600
O1···N2	3.017 (3)	H12···S1^ii^	2.9300
O1···N1^x^	2.829 (3)	H13···F6A	2.7500
O1···N3	2.716 (3)	H13···F4A	2.8500
O1···H3A	2.0100	H13···S1^ii^	3.1000
O1···H1^x^	1.9800	H13···F4B	2.3300
N1···O1^x^	2.829 (3)	H13···F5C	2.4200
N1···C12^vi^	3.447 (4)	H15···F6C	2.5500
N1···S1^xi^	3.524 (3)	H15···F6B	2.5900
N2···O1	3.017 (3)	H15···F5A	2.4400
N2···N4	2.627 (3)	H16···F1A^ix^	2.7900
N2···F4C^vi^	3.19 (2)	H16···F1B^ix^	2.7600
N2···F6A^vi^	3.045 (8)	H16···H4A	2.2600
			
C5—O2—C9	115.9 (2)	N4—C11—C16	116.8 (2)
C1—N1—C2	111.5 (2)	N4—C11—C12	124.2 (2)
N3—N2—C8	116.3 (2)	C11—C12—C13	119.3 (2)
N2—N3—C10	122.3 (2)	C12—C13—C14	121.4 (2)
C10—N4—C11	131.0 (2)	C13—C14—C15	119.2 (2)
C2—N1—H1	124.00	C13—C14—C17	119.8 (2)
C1—N1—H1	124.00	C15—C14—C17	121.0 (3)
N2—N3—H3A	119.00	C14—C15—C16	120.1 (3)
C10—N3—H3A	119.00	C11—C16—C15	120.9 (2)
C10—N4—H4A	114.00	F4A—C17—C14	113.1 (4)
C11—N4—H4A	114.00	F4A—C17—F5A	105.1 (6)
O1—C1—N1	126.9 (2)	F4A—C17—F6A	104.2 (4)
N1—C1—C8	106.3 (2)	F5C—C17—C14	111.4 (10)
O1—C1—C8	126.8 (2)	F6B—C17—C14	108.0 (8)
N1—C2—C7	109.2 (2)	F6C—C17—C14	112.3 (9)
N1—C2—C3	128.5 (2)	F4B—C17—F5B	107.0 (15)
C3—C2—C7	122.3 (2)	F4B—C17—F6B	103.6 (16)
C2—C3—C4	117.7 (2)	F4C—C17—F5C	106.9 (15)
C3—C4—C5	119.9 (2)	F4C—C17—F6C	107.7 (18)
C4—C5—C6	123.2 (2)	F5B—C17—F6B	116.9 (16)
O2—C5—C4	118.6 (2)	F5C—C17—F6C	106.5 (15)
O2—C5—C6	118.1 (2)	F5A—C17—F6A	106.0 (6)
C5—C6—C7	116.9 (2)	F5A—C17—C14	115.0 (3)
C2—C7—C8	106.7 (2)	F6A—C17—C14	112.5 (4)
C6—C7—C8	133.3 (2)	F4B—C17—C14	109.2 (8)
C2—C7—C6	120.0 (2)	F4C—C17—C14	111.7 (12)
C1—C8—C7	106.2 (2)	F5B—C17—C14	111.7 (9)
N2—C8—C1	127.0 (2)	C2—C3—H3	121.00
N2—C8—C7	126.9 (2)	C4—C3—H3	121.00
F1A—C9—F3	100.9 (7)	C3—C4—H4	120.00
F1A—C9—F2	109.0 (4)	C5—C4—H4	120.00
F1B—C9—O2	97.3 (12)	C5—C6—H6	122.00
F1A—C9—O2	117.2 (5)	C7—C6—H6	122.00
F2—C9—F3	107.5 (3)	C11—C12—H12	120.00
F2—C9—O2	109.1 (3)	C13—C12—H12	120.00
F1B—C9—F2	100.0 (11)	C12—C13—H13	119.00
F3—C9—O2	112.6 (3)	C14—C13—H13	119.00
F1B—C9—F3	128.7 (14)	C14—C15—H15	120.00
S1—C10—N3	117.20 (18)	C16—C15—H15	120.00
S1—C10—N4	129.66 (19)	C11—C16—H16	120.00
N3—C10—N4	113.1 (2)	C15—C16—H16	120.00
C12—C11—C16	119.0 (2)		
			
C9—O2—C5—C4	88.2 (3)	C2—C3—C4—C5	−0.5 (4)
C9—O2—C5—C6	−95.3 (3)	C3—C4—C5—O2	178.3 (2)
C5—O2—C9—F1A	−58.3 (7)	C3—C4—C5—C6	1.9 (4)
C5—O2—C9—F2	177.3 (2)	O2—C5—C6—C7	−177.7 (2)
C5—O2—C9—F3	58.1 (4)	C4—C5—C6—C7	−1.3 (4)
C2—N1—C1—O1	176.1 (2)	C5—C6—C7—C2	−0.6 (3)
C2—N1—C1—C8	−2.7 (3)	C5—C6—C7—C8	−178.4 (2)
C1—N1—C2—C3	−178.1 (2)	C2—C7—C8—N2	177.6 (2)
C1—N1—C2—C7	1.2 (3)	C2—C7—C8—C1	−2.6 (3)
C8—N2—N3—C10	179.4 (2)	C6—C7—C8—N2	−4.4 (4)
N3—N2—C8—C1	−1.0 (4)	C6—C7—C8—C1	175.4 (3)
N3—N2—C8—C7	178.8 (2)	N4—C11—C12—C13	−177.5 (3)
N2—N3—C10—S1	176.33 (19)	C16—C11—C12—C13	2.9 (4)
N2—N3—C10—N4	−3.3 (3)	N4—C11—C16—C15	177.6 (2)
C11—N4—C10—S1	−8.8 (4)	C12—C11—C16—C15	−2.7 (4)
C11—N4—C10—N3	170.8 (2)	C11—C12—C13—C14	−0.6 (4)
C10—N4—C11—C12	15.8 (4)	C12—C13—C14—C15	−1.9 (4)
C10—N4—C11—C16	−164.5 (2)	C12—C13—C14—C17	176.8 (3)
O1—C1—C8—N2	4.2 (4)	C13—C14—C15—C16	2.1 (4)
O1—C1—C8—C7	−175.6 (2)	C17—C14—C15—C16	−176.6 (3)
N1—C1—C8—N2	−176.9 (2)	C13—C14—C17—F4A	−61.2 (5)
N1—C1—C8—C7	3.3 (3)	C13—C14—C17—F5A	178.1 (6)
N1—C2—C3—C4	177.7 (2)	C13—C14—C17—F6A	56.5 (5)
C7—C2—C3—C4	−1.5 (4)	C15—C14—C17—F4A	117.4 (5)
N1—C2—C7—C6	−177.3 (2)	C15—C14—C17—F5A	−3.3 (6)
N1—C2—C7—C8	1.0 (3)	C15—C14—C17—F6A	−124.9 (4)
C3—C2—C7—C6	2.1 (4)	C14—C15—C16—C11	0.2 (4)
C3—C2—C7—C8	−179.7 (2)		

Symmetry codes: (i) *x*, *y*−1, *z*; (ii) −*x*+1, −*y*+1, −*z*+1; (iii) −*x*+1, −*y*+2, −*z*+1; (iv) −*x*+1, −*y*+1, −*z*; (v) −*x*, −*y*+1, −*z*; (vi) *x*+1, *y*+1, *z*; (vii) *x*−1, *y*−2, *z*; (viii) *x*−1, *y*−1, *z*; (ix) −*x*+1, −*y*+2, −*z*; (x) −*x*+2, −*y*+3, −*z*+1; (xi) *x*, *y*+1, *z*; (xii) *x*+1, *y*+2, *z*.

### Hydrogen-bond geometry (Å, °)

**Table:**

*D*—H···*A*	*D*—H	H···*A*	*D*···*A*	*D*—H···*A*
N1—H1···O1^x^	0.8600	1.9800	2.829 (3)	168.00
N3—H3A···O1	0.8600	2.0100	2.716 (3)	138.00
N4—H4A···N2	0.8600	2.1900	2.627 (3)	111.00
C12—H12···S1	0.9300	2.5600	3.210 (3)	128.00
C15—H15···F5A	0.9300	2.4400	2.763 (6)	100.00

Symmetry codes: (x) −*x*+2, −*y*+3, −*z*+1.
